# Highly Sensitive and Real-Time Detection of Zinc Oxide Nanoparticles Using Quartz Crystal Microbalance via DNA Induced Conjugation

**DOI:** 10.3390/ma15176113

**Published:** 2022-09-02

**Authors:** Chanho Park, Hyunjun Park, Juneseok You, Sungsoo Na, Kuewhan Jang

**Affiliations:** 1 Division of Foundry, Samsung Electronics, Hwaseong-si 18448, Korea; 2 Department of Mechanical Engineering, Korea University, Seoul 02841, Korea; 3 School of Mechanical and Automotive Engineering, Hoseo University, Asan 31499, Korea

**Keywords:** zinc oxide nanoparticles, quartz crystal microbalance, DNA, high-sensitive, real-time detection, conjugation

## Abstract

With the development of nanotechnology, nanomaterials have been widely used in the development of commercial products. In particular, zinc oxide nanoparticles (ZnONPs) have been of great interest due to their extraordinary properties, such as semiconductive, piezoelectric, and absorbance properties in UVA and UVB (280–400 nm) spectra. However, recent studies have investigated the toxicity of these ZnONPs; therefore, a ZnONP screening tool is required for human health and environmental problems. In this study, we propose a detection method for ZnONPs using quartz crystal microbalance (QCM) and DNA. The detection method was based on the resonance frequency shift of the QCM. In detail, two different complementary DNA strands were used to conjugate ZnONPs, which were subjected to mass amplification. One of these DNA strands was designed to hybridize to a probe DNA immobilized on the QCM electrode. By introducing the ZnONP conjugation, we were able to detect ZnONPs with a detection limit of 100 ng/mL in both distilled water and a real sample of drinking water, which is 3 orders less than the reported critical harmful concentration of ZnONPs. A phosphate terminal group, which selectively interacts with a zinc oxide compound, was also attached at one end of a DNA linker and was attributed to the selective detection of ZnONPs. As a result, better selective detection of ZnONPs was achieved compared to gold and silicon nanoparticles. This work demonstrated the potential of our proposed method as a ZnONP screening tool in real environmental water systems.

## 1. Introduction

In recent years, there has been rapid growth in the use of nanomaterials, from scientific research to commercial products. Commercial products, such as sun creams, tennis rackets, solid lubricants, and detergents, contain nanomaterials due to their extraordinary properties, which cannot be observed in the bulk state [1]. Among nanomaterials, zinc oxide nanoparticles (ZnONPs) are one of the most widely used nanomaterials due to their piezoelectric, semiconductor, and absorbance in UVA and UVB (280–400 nm) spectra [2].

Although ZnONPs are widely used in commercial products, human exposure to ZnONPs has not been studied. Effluents from manufacturing, consumer utilization, and disposal of ZnONPs increase their release into aquatic systems and eventually increase human exposure to ZnONPs [3,4]. Recent studies have investigated the toxicity effect of ZnONPs [5,6]. Although zinc is a critically important trace element for several biological functions and is known to be relatively harmless, rapid ionization can subsequently occur in cells that are in a weak acidic state [7]. Due to the high surface-to-volume ratio of ZnONPs, excess zinc ion secretion can occur, which can lead to a cytotoxic phenomenon, and the critical harmful concentration is approximately 10 μg/mL of ZnONPs in a weak acid solution [8].

The development of a ZnONP screening tool is very important for human health and environmental problems. For toxicity assessments of ZnONPs in relation to water pollution, environmental hazards, and human health, sensitive screening tools are required [1]. Numerous approaches have been used for the detection of ZnONPs, using a scanning electron microscope (SEM) [9], transmission electron microscope (TEM) [10], and inductively coupled plasma-mass spectrometry (ICP-MS) [11], etc. These approaches are more effective as methods for characterization rather than detection because they require a confined laboratory environment for an operation. The screening of ZnONPs should not be confined to a laboratory environment, and no research effort has yet been made for its efficient and rapid detection.

In this study, we propose a detection method for ZnONPs using quartz crystal microbalance (QCM) and DNA. The detection mechanism is based on the resonance frequency shift of QCM upon the adsorption of a ZnONP conjugation on the QCM electrode. Two complementary DNA strands were used to conjugate ZnONPs, which were subjected to mass amplification. One of these DNA strands was designed to hybridize to a probe DNA (p-DNA), which was immobilized on the QCM electrode prior to detection. When the solution that contained ZnONP conjugation was exposed to the QCM electrode, the conjugation adsorbed on the QCM electrode, and the mass of the electrode increased. As a result, a frequency shift was observed [12], and by evaluating the shift, we were able to detect ZnONPs in both distilled (DI) and commercially available drinking water with the limit of detection (LOD) of 100 ng/mL. The phosphate terminal group, which selectively interacts with the zinc oxide compound, leads to the selective detection of ZnONPs [13]. As a result, selective detection of ZnONPs was achieved.

## 2. Materials and Methods

### 2.1. Materials

The following materials were purchased from Sigma-Aldrich, Merck Corporation (St. Louis, MO, USA): zinc oxide nanoparticles (ZnONPs), gold nanoparticles (AuNPs), silicon oxide nanoparticles (SiO_2_NPs), sodium chloride (NaCl), sulfuric acid (H_2_SO_4_), hydrogen peroxide (H_2_O_2_), and tris-ethylenediaminetetraacetic acid (Tris-EDTA) buffer solution. Single-stranded DNA (ssDNA) strands were purchased from Integrated DNA Technology (Coralville, IA, USA): 5′-/5Phos/GGG GGG GTT GCG AGG TCT TGC CGA CA-3′ (l1-DNA), 5′-/5Phos/GGG GGG TGT CGG CAA GAC CTC GCA AC-3′ (l2-DNA), and 5′-/5ThioMC6-D/GGG GGG TGT CGG CAA GAC CTC GCA AC-3′ (p-DNA). Each DNA strand was dissolved in Tris-EDTA buffer solution, and the solution was refrigerated (~4 °C) for further use. All DNA strands were composed of a spacer (6 mer poly-G) and a 20 mer recognition sequence for DNA hybridization to reduce the steric crowding and increase the accessibility of the nucleotide bases for DNA hybridization [14,15]. The recognition sequence was designed specifically to prevent secondary structure using the IDT OligoAnalyzer™ Tool (www.idtdna.com/pages/tools//oligoanalyzer).

### 2.2. Synthesis of ZnONP Conjugation, SiO_2_NPs, and AuNP Solutions

The ZnONPs were placed in distilled (DI) water, and the concentration of ZnONPs was adjusted during the process. Two different complementary DNA strands (l1-DNA and l2-DNA) were used to conjugate the ZnONPs. These DNA strands could hybridize each other, and also bind to the outer surface of the ZnONPs by a phosphate terminal group [13] that is attached at both ends of the hybridized DNA. Consequently, the phosphorus atom of the DNA terminal group and the hydroxy groups of the ZnONP surface form strong covalent P-O-Zn anchoring. In this manner, the DNA strands could act as linkers between the ZnONPs, producing ZnONP conjugation. In detail, Linker DNA strands were added to the bare ZnONP solution, and the concentrations of l1-DNA and l2-DNA were 36 μM and 33 μM, respectively, similar to previous reports [16,17]. As l1-DNA was hybridized with both l2-DNA and p-DNA, l1-DNA was added at 10% excess to l2-DNA so that a remainder of l1-DNA existed that could hybridize to p-DNA. The solution was mixed for 16 h, and the mixtures were kept in a refrigerator (4 °C) for further use. SiO_2_NP and AuNP solutions were synthesized similarly. A X-ray photoelectron spectroscopy (XPS) and UV-vis spectrum were obtained using a ULVAC-PHI, X-TOOL, Inc., Kanagawa, Japan, and UV/Vis spectrophotometer (Hach, DR-4000), respectively.

### 2.3. Probe DNA Immobilization on the QCM Electrode

We immobilized DNA denoted as p-DNA on the QCM electrode, where the DNA was designed to hybridize with l1-DNA. The p-DNA was similar to l2-DNA, except that the DNA contained a thiol terminal group instead of a phosphate terminal group. In detail, the QCM electrode was cleaned with a piranha solution (3:1 mixture of H_2_SO_4_ and H_2_O_2_) and then rinsed thoroughly with DI water. The electrode was then dried using N_2_ gas, sterilized in UV light, and stored in a desiccator. For DNA immobilization, 200 μL of p-DNA solution (10 μM of p-DNA and 10 μM of NaCl) was continuously dropped onto the electrode for a period of 3 h to facilitate covalent bonding of the modified thiol group to the gold surface of the electrode. After 3 h, the QCM electrode was washed again with DI water to remove the physical attachments.

### 2.4. Detection of ZnONPs

The QCM electrode was mounted in a flow cell of the QCM instrument (Stanford Research Systems, Sunnyvale, CA, USA) for real-time detection. To avoid the liquid damping effect of QCM, DI water was flowed (flow rate of 0.15 mL/s) to the QCM flow to measure the baseline reference points. Once the resonance frequency for the reference point was stabilized, detection was performed by switching the flow of the solution from DI water to the solution containing ZnONP conjugation. The flow rate of the ZnONP conjugation solution was also set at 0.15 mL/s. The detection was conducted for 1 h in a controlled constant room temperature (~22 °C) environment. The concentrations of ZnONPs used in the experiments ranged from 10 μg/mL to 10 ng/mL. The change in the resonance frequency of the QCM was measured using SRS QCM200 LabVIEW 2.0 software (National Instruments Corporation, Austin, TX, USA). Detection was also performed using a real sample of drinking water. In a simple way, probe DNA with both thiol and phosphate terminal groups at each end could be used to detect ZnONPs directly, without using linker DNA. However, by this approach, ZnONP conjugation could not be formed, so the detection signal would be decreased and would not be suitable for highly sensitive detection.

### 2.5. Atomic Force Microscopy Analysis

The surface morphology of the bare ZnONPs and the ZnONP conjugation were observed using atomic force microscopy (AFM). Each ZnONP sample was deposited on a silicon wafer via physical adsorption. The surface of each wafer was scanned using AFM “tapping” mode with a TESPA cantilever probe (Bruker, Santa Barbara, CA, USA). Images were obtained using a nanodrive controller (Bruker, Santa Barbara, CA, USA). The dimensions of the scan area were 5 × 5 μm, and the scan speed was set at 1 Hz. The recorded data were analyzed using SPM Lab Analysis software V1.20 (Bruker, Santa Barbara, CA, USA).

## 3. Results

### 3.1. Detection Overview

**Figure 1:**
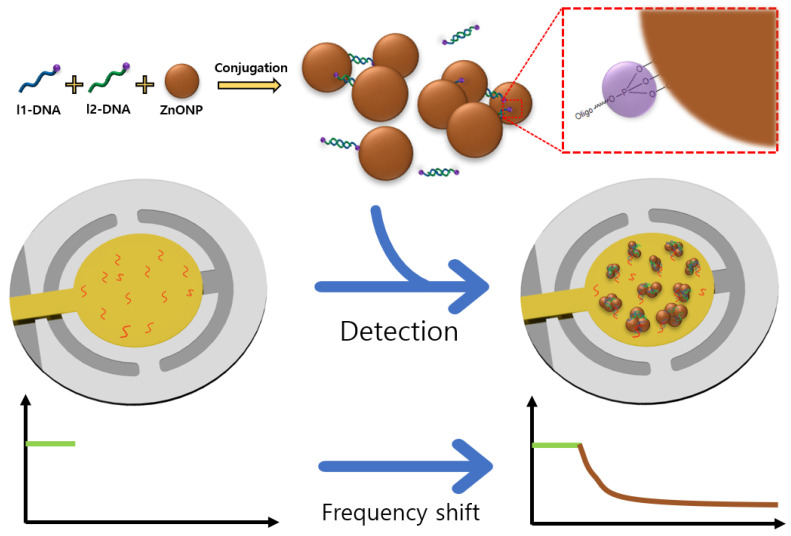
Schematic illustration of the ZnONP detection method using quartz crystal microbalance (QCM) and DNA.

For ZnONP detection, we used a QCM that has been employed for highly sensitive and real-time detection of various elements, such as nanomaterials, gas molecules, and the bio-recognition of elements [18,19,20]. We also used 2 different DNA linkers to conjugate ZnONPs (Figure 1). The advantage of ZnONP conjugation is mass amplification due to the increased number of ZnONPs, as well as the additional DNA that remained in ZnONP conjugation, which eventually attributed to the highly sensitive detection [17]. When the ZnONP conjugation solution flowed on the p-DNA immobilized electrode, ZnONP conjugation bound to the electrode by DNA hybridization between the l1-DNA of the ZnONP conjugation and the p-DNA of the electrode. From the Sauerbrey equation [12], it is well known that the resonance frequency shift of a QCM is attributed to mass variation. The binding of ZnONP conjugation to the QCM electrode increased its mass and led to a resonance frequency shift. ZnONP detection was achieved by evaluating the frequency shift in real-time.

### 3.2. Verification of ZnONP Conjugation

**Figure 2:**
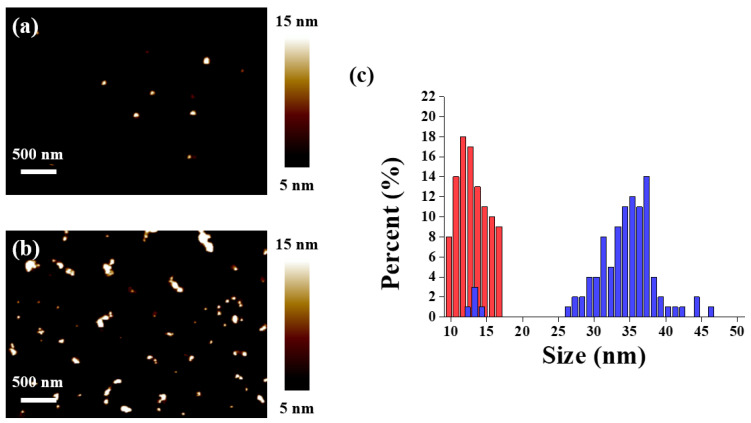
Tapping-mode AFM images of (**a**) bare (un-conjugated) and (**b**) conjugated ZnONPs. (**c**) Height distribution of bare (red) and conjugated (blue) ZnONPs.

To verify ZnONP conjugation, we deposited the bare (un-conjugated) and ZnONP conjugated samples on a silicon wafer and obtained the morphologies using atomic force microscopy (AFM). AFM images showed typical single-particle morphology for the bare ZnONP samples and particle-cluster morphology for the ZnONP conjugation sample. (Figure 2a,b). For further verification, we evaluated approximately 100 ZnONPs and ZnONP clusters and measured the height of each structure (Figure 2c). The height of bare ZnONPs was in the range of 10–17 nm, whereas the height of ZnONP conjugation was mostly in the range of 26–46 nm. However, in the ZnONP conjugation sample, we also observed a small portion of particles in the height range of 12–14 nm. This result might be due to the un-conjugated ZnONP remainder. Theoretically, a single ZnONP adsorbed by DNA linkers could also exist. If the case occurred, the height would be in the range of 23.6–30.6 nm at most, taking into account the contribution of the 20 base pair (bp) DNA duplex height as 6.8 nm (0.34 nm per base pair) at both sides. However, the calculated height range was significantly smaller than that of the ZnONP conjugation. The significant increase in height was indicative of ZnONP conjugation.

We also observed a solution of bare ZnONPs (sample without linker DNA strands) and ZnONP conjugation (sample with linker DNA strands) after 48 h of production (Figure S1a). In the case of the bare solution, significant sediment was observed, whereas the ZnONP conjugation solution remained well-dispersed. This result implies that the linker DNA was well bound to the surface of ZnONPs due to the negatively charged backbone of DNA that stabilizes ZnONPs in the solution [21].

UV-vis spectra of the bare ZnONPs and ZnONP conjugation solutions were obtained. The major peaks of bare ZnONPs and ZnONP conjugation were obtained at 366 and 367 nm, respectively (Figure S2). A slight peak shift (1 nm) of the ZnONP conjugation spectra was observed compared to that of the bare ZnONP spectra. The peak shift was due to the covalent anchoring between DNA linkers and ZnONPs, similar to that seen in a previous study [22].

For further verification, we obtained XPS (X-ray photoelectron spectroscopy) spectra of the bare ZnONPs, ZnONP conjugation, and linker DNA samples. We observed significant Zn2p_1/2_, Zn2p_3/2_, O1s, Zn LMM, Zn3s, Zn3p, and Zn3d peaks from the bare ZnONPs and ZnONP conjugation spectra, indicating the existence of ZnONPs (Figure S3a). Significant C1s and O1s peaks were observed from the linker DNA sample, as expected (Figure S3a). In the case of the O1s peak from the ZnONPs and ZnoNP conjugation spectra, the O1s peaks can be fit by two Voigt peaks at ~531.5 and ∼530.5 eV. The 531.5 eV peak arises from hydroxyl groups and grafted phosphonic acid molecules, and the 530.5 eV peak is attributed to O atoms in the underlying bulk ZnO [23]. The increase in the relative intensity of the 531.5 eV peak in the ZnONP conjugation spectra is evidence of P-O-Zn anchoring between the linker DNA and the ZnONPs (Figure S3b). Phosphonic acid modifications were identified by evaluating the P2p binding energy of 133.6 eV [24]. A significant P2p peak was observed only in the ZnONP conjugation spectra, and the result also indicates P-O-Zn anchoring (Figure S3c).

### 3.3. Real-Time and Quantitative Detection of ZnONPs

For ZnONP detection, p-DNA with a thiol terminal group was immobilized on the chrome/gold QCM electrode through gold–thiol bonding [25]. To verify the p-DNA immobilization on the electrode surface, the surface morphology of the bare electrode and the p-DNA immobilized electrode were compared using AFM. The AFM images (Figure S4a,b) revealed that the overall height of the p-DNA immobilized electrode increased compared to the bare electrode. The arithmetic average (Ra) value of the surface roughness was calculated for each sample. The Ra values of the bare electrode and the p-DNA-immobilized electrode were 0.33 ± 0.07 nm and 0.52 ± 0.09 nm, respectively (Figure S4c). The increase in Ra value was a result of p-DNA immobilization on the electrode surface, and the small standard deviation in the measurements was due to the uniform deposition of p-DNA.

**Figure 3:**
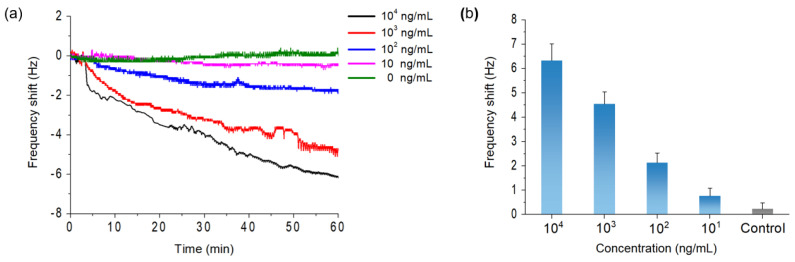
(**a**) Real-time detection and (**b**) frequency shift after 1 h with respect to ZnONP concentrations of 10^4^, 10^3^, 10^2^, 10 and 0 ng/mL (control) in DI water.

For the quantitative detection of ZnONPs, a ZnONP solution was prepared with concentrations of 10^4^, 10^3^, 10^2^, 10, and 0 (control) ng/mL. The ZnONP solutions with concentrations of 10^6^ and 10^5^ ng/mL showed whitish color, which was significantly recognized by the naked eye (data not shown). The solutions with concentrations below 10^4^ ng/mL were transparent, similar to the bare drinking water (data not shown). Therefore, we chose 10^4^ ng/mL as the highest concentration for the experiment. For the control experiment, we prepared a solution with l1-DNA and l2-DNA without ZnONPs. We also detected ZnONPs with a bare electrode (without p-DNA). A frequency shift was not observed (data not shown). The p-DNA immobilized electrode was mounted onto the QCM flow cell, and the control solution (DI water) was flowed through the flow cell. A sudden drop in the frequency shift occurred (data not shown) due to the liquid loading effect [26], and then the resonance frequency stabilized. Once the frequency was stabilized, detection was performed by switching the flow from the control solution to the solution containing ZnONPs. As shown in Figure 3a, the real-time frequency shift of the QCM was monitored with different ZnONP concentrations. The frequency shift occurred constantly, and a higher frequency shift was observed in higher concentrations of ZnONPs. For further verification, a repeat experiment was conducted, and the frequency shift values after 1 h were evaluated, as shown in Figure 3b. The frequency shifts at the concentrations of 10^4^, 10^3^, 10^2^, 10, and 0 ng/mL were 6.33 ± 0.68, 4.55 ± 0.49, 2.13 ± 0.40, 0.77 ± 0.32, and 0.23 ± 0.25 Hz, respectively. A greater frequency shift was observed at higher concentrations of ZnONPs. In the case of the 10 ng/mL concentration, a significant difference in the frequency shift relative to the control sample was not observed. However, the frequency shift at 10^2^ ng/mL concentration was approximately 8.5 times greater than the standard deviation of the control sample, and this result implies that the sensitivity of LOD was 10^2^ ng/mL. The obtained LOD was 100 times less than the reported critical harmful concentration of ZnONPs [8]; therefore, highly sensitive detection was achieved.

**Figure 4:**
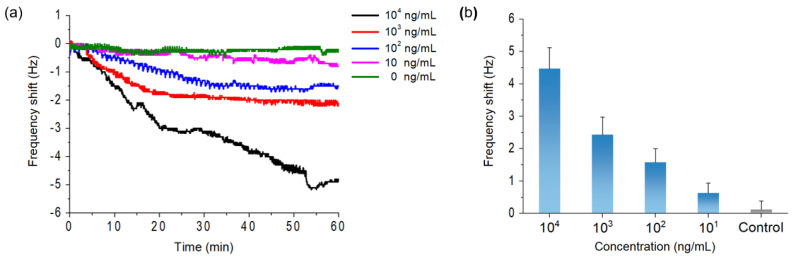
(**a**) Real-time detection and (**b**) frequency shift after 1 h with respect to ZnONP concentrations of 10^4^, 10^3^, 10^2^, 10 and 0 ng/mL (control) in drinking water.

To validate our proposed method as an effective ZnONP screening tool in real environmental samples, the detection ability in either drinking or tap water should be confirmed. Since the most common human exposure route to ZnONPs is ingestion rather than skin penetration (ZnONPs are not able to penetrate beyond the stratum corneum of skin) [27], and the breakthrough of ZnONPs into treated drinking water is a major issue [28], we performed additional detection experiments using commercially available drinking water (SamDaSoo, Kwangdong Corp., Korea) instead of tap water. Figure 4a shows the real-time detection results of ZnONPs in drinking water, and a frequency shift was observed constantly. The observed frequency shifts were 4.47 ± 0.65, 2.43 ± 0.55, 1.58 ± 0.42, 0.63 ± 0.31, and 0.12 ± 0.27 Hz for ZnONP concentrations of 10^4^, 10^3^, 10^2^, 10, and 0 ng/mL, respectively (Figure 4b). Similar to the detection in DI water, the overall frequency shift value decreased with a decrease in ZnONP concentration, and the LOD in drinking water was evaluated as 100 ng/mL. The LOD in both the DI and drinking water was 100 ng/mL; however, the overall frequency shift value in the drinking water was lower than the value from the DI water. This phenomenon is likely due to interference from non-specific binding with various substances present in drinking water [29].

### 3.4. Selective Detection of ZnONPs

An efficient ZnONP screening tool should be capable of selective detection. The selective detection of ZnONPs was evaluated as opposed to other types of nanoparticles. SiO_2_NPs and AuNPs were chosen for comparison due to their wide real-world applications (Figure S1b). The concentration of each particle was 10^4^ ng/mL. For convenience, the detection result was evaluated using a relative frequency shift obtained using the following equation:

Relative frequency shift (%) = 100 × Δf_other_/(Δf_ZnONP_); (1)

where Δf_other_ and Δf_ZnONP_ were the frequency shifts for the tested SiO_2_NPs or AuNPs, and ZnONPs, respectively.

**Figure 5:**
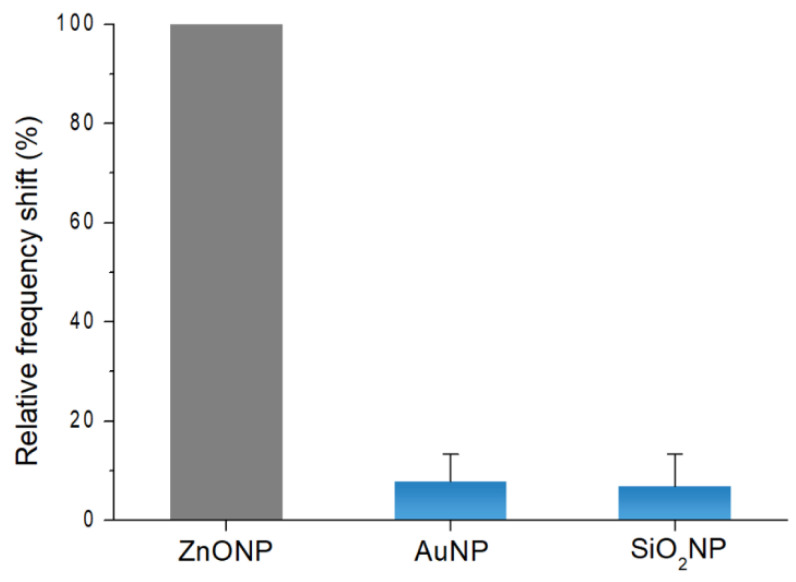
Detection results of AuNPs and SiO_2_NPs relative to the frequency shift of ZnONPs. The concentration of all nanoparticles was 10^4^ ng/mL.

The relative frequency shifts of SiO_2_NP and AuNP detection were 7.90 ± 5.47 and 6.85 ± 6.58 percent, respectively (Figure 5). SiO_2_NPs and AuNPs displayed significantly low-frequency shift values (<10%), which were negligible in comparison to the ZnONP detection values. The results were due to the specific interactions between the outer surface of ZnONPs and the phosphate terminal group of linker DNA, which could not occur between the silicon oxide and gold surfaces.

## 4. Conclusions

In summary, we proposed a highly sensitive and real-time detection method for ZnONPs using QCM and DNA. The detection was based on the resonance frequency shift of QCM upon the adsorption of ZnONPs and DNA on the QCM electrode. Highly sensitive detection was achieved by introducing ZnONP conjugation using DNA linkers, which were subjected to mass amplification. As a result, the sensitivity of the LOD was 100 ng/mL, which was 3 orders lower than the reported ZnONP toxicity concentration [8]. The phosphate terminal group of the linker DNA, which selectively interacted with the zinc oxide surface, was responsible for the selective detection of ZnONPs, as opposed to SiO_2_NP and AuNP. Finally, our proposed method was able to detect ZnONPs even in real environmental samples of commercially available drinking water. The performance of our proposed method has possible applications as a ZnONP screening tool in real environmental water systems.

## Data Availability

Not applicable.

## Supplementary Materials

The following supporting information can be downloaded at: https://www.mdpi.com/article/10.3390/ma15176113/s1, Figure S1: QCM electrode AFM analysis images; Figure S2: UV-visible spectra; Figure S3: XPS spectra; Figure S4: Optical images.
