# Supplementary material for: Highly Sensitive and Real-Time Detection of Zinc Oxide Nanoparticles Using Quartz Crystal Microbalance via DNA Induced Conjugation
Source: Materials (Basel). 2022 Sep 2;15(17):6113. doi: 10.3390/ma15176113 (PMC9458006; doi:10.3390/ma15176113)
Supplement: Supplementary file 1 [file materials-15-06113-s001.zip › materials-1833628-supplementary.pdf]

materials

MDPI

Article

Highly Sensitive and Real-Time Detection of Zinc Oxide Nanoparticles Using Quartz Crystal Microbalance via DNA Induced Conjugation

Chanho Park [1,†], Hyunjun Park [2,†], Juneseok You [2], Sungsoo Na [2,*] and Kuewhan Jang [3,*]

1 Division of Foundry, Samsung Electronics, Hwaseong-si 18448, Korea
2 Department of Mechanical Engineering, Korea University, Seoul 02841, Korea
3 School of Mechanical and Automotive Engineering, Hoseo University, Asan 31499, Korea
† These authors contributed equally to this work.
* Correspondence: nass@korea.ac.kr (S.N.); kwjang@hoseo.edu (K.J.)

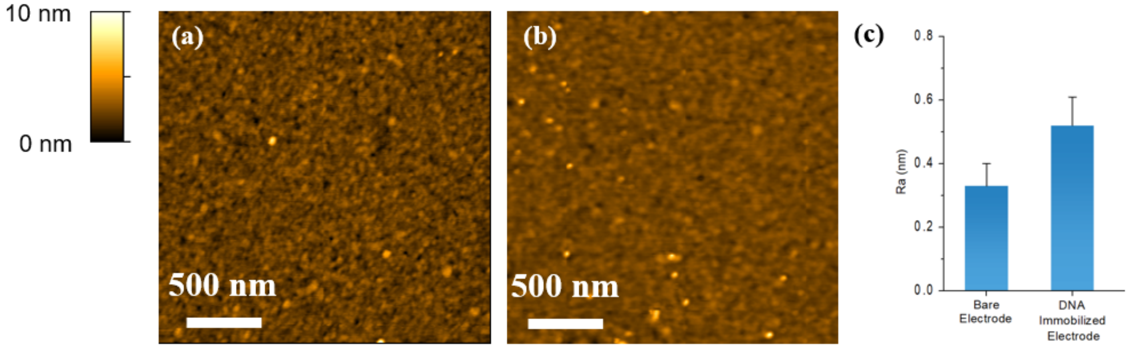

Figure S1. Tapping-mode AFM images of (**A**) bare and (**B**) DNA immobilized electrodes. The scale bar is 500 nm. (**C**) Surface roughness of bare and DNA immobilized electrodes.

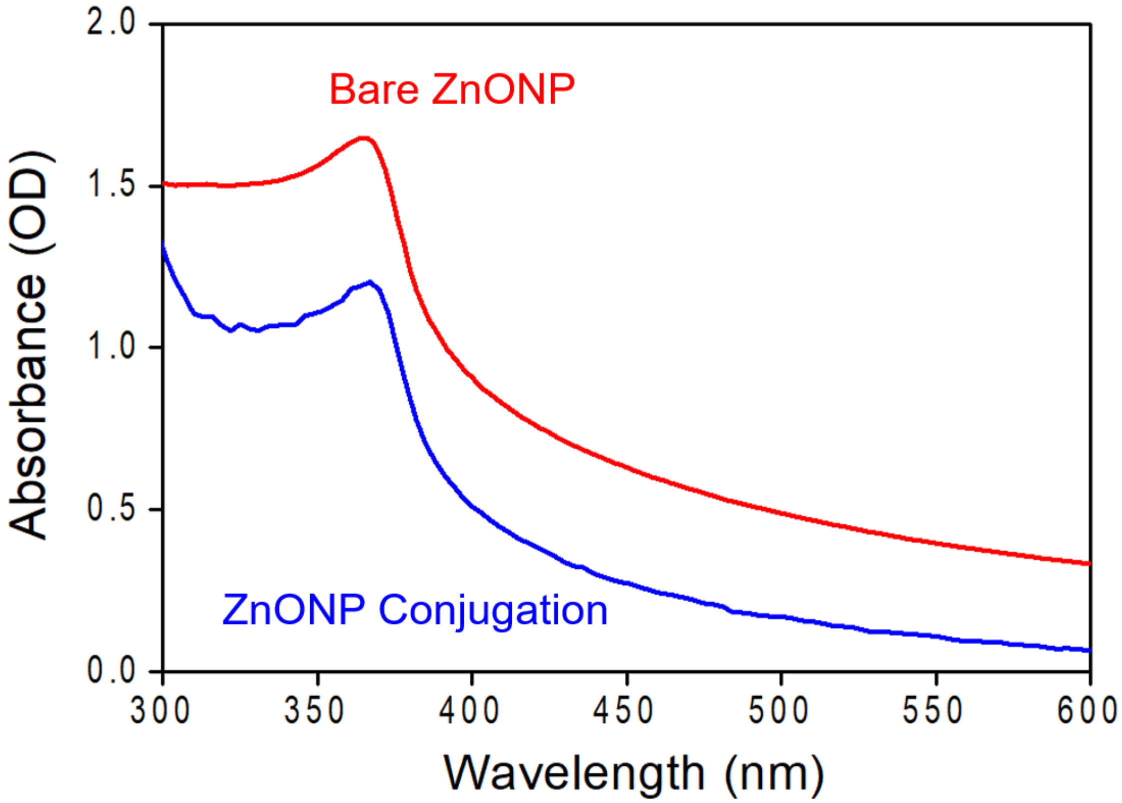

Figure S2. UV-visible spectra of ZnO NP(red) and ZnONP conjugation(blue). The peak of ZnO NP and ZnONP conjugation appear at 369 and 370 nm, respectively.

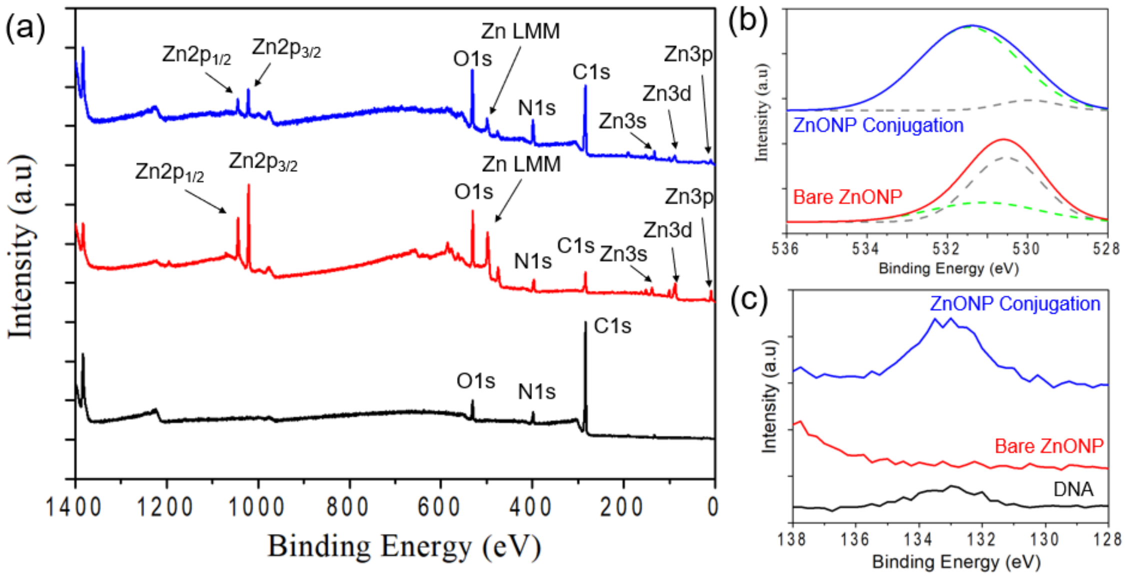

Figure S3. (**a**) Wide scan, (**b**) O1s and (**c**) P2p XPS spectra of DNA(black), bare ZnONP(red), and ZnONP conjugation(blue).

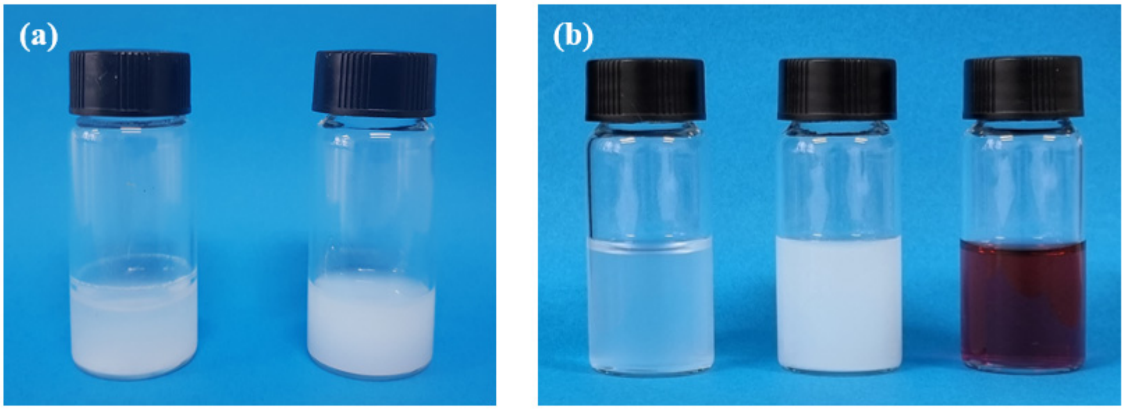

Figure S4. (**a**) Optical image of bare ZnONP(left) and ZnONP conjugation(right) solutions after 48 hours of production. (**b**) Optical image of SiO2NP(left), ZnONP(middle) and AuNP(right) solutions.
